# Systematic review of the prospective association of daily step counts with risk of mortality, cardiovascular disease, and dysglycemia

**DOI:** 10.1186/s12966-020-00978-9

**Published:** 2020-06-20

**Authors:** Katherine S. Hall, Eric T. Hyde, David R. Bassett, Susan A. Carlson, Mercedes R. Carnethon, Ulf Ekelund, Kelly R. Evenson, Deborah A. Galuska, William E. Kraus, I-Min Lee, Charles E. Matthews, John D. Omura, Amanda E. Paluch, William I. Thomas, Janet E. Fulton

**Affiliations:** 1 Geriatric Research, Education, and Clinical Center, Durham VA Health Care System, Durham, NC USA; 2 Claude D. Pepper Older Americans Independence Center, Duke Aging Center, and the Department of Medicine, Duke University, Durham, NC USA; 3 Division of Nutrition, Physical Activity, and Obesity, National Center for Chronic Disease Prevention and Health Promotion, Centers for Disease Control and Prevention, Atlanta, GA USA; 4 Department of Kinesiology, Recreation, and Sport Studies, The University of Tennessee, Knoxville, TN USA; 5 Department of Preventive Medicine, Northwestern University, Chicago, IL USA; 6 Department of Sport Medicine, Norwegian School of Sport Sciences, Oslo, Norway and Department of Chronic Diseases and Ageing, Norwegian Institute of Public Health, Oslo, Norway; 7 Department of Epidemiology, Gillings School of Global Public Health, University of North Carolina – Chapel Hill, Chapel Hill, NC USA; 8 Brigham and Women’s Hospital, Harvard Medical School; Harvard T.H. Chan School of Public Health, Boston, MA USA; 9 Metabolic Epidemiology Branch, Division of Cancer Epidemiology and Genetics, National Cancer Institute, Rockville, MD USA; 10 Department of Kinesiology, Institute for Applied Life Sciences, University of Massachusetts, Amherst, MA USA; 11 Office of Library Science, Office of Science, Centers for Disease Control and Prevention, Atlanta, GA USA

**Keywords:** Physical activity, Walking, Diabetes, Prevention, Accelerometer, Physical activity guidelines, Public health

## Abstract

**Background:**

Daily step counts is an intuitive metric that has demonstrated success in motivating physical activity in adults and may hold potential for future public health physical activity recommendations. This review seeks to clarify the pattern of the associations between daily steps and subsequent all-cause mortality, cardiovascular disease (CVD) morbidity and mortality, and dysglycemia, as well as the number of daily steps needed for health outcomes.

**Methods:**

A systematic review was conducted to identify prospective studies assessing daily step count measured by pedometer or accelerometer and their associations with all-cause mortality, CVD morbidity or mortality, and dysglycemia (dysglycemia or diabetes incidence, insulin sensitivity, fasting glucose, HbA1c). The search was performed across the Medline, Embase, CINAHL, and the Cochrane Library databases from inception to August 1, 2019. Eligibility criteria included longitudinal design with health outcomes assessed at baseline and subsequent timepoints; defining steps per day as the exposure; reporting all-cause mortality, CVD morbidity or mortality, and/or dysglycemia outcomes; adults ≥18 years old; and non-patient populations.

**Results:**

Seventeen prospective studies involving over 30,000 adults were identified. Five studies reported on all-cause mortality (follow-up time 4–10 years), four on cardiovascular risk or events (6 months to 6 years), and eight on dysglycemia outcomes (3 months to 5 years). For each 1000 daily step count increase at baseline, risk reductions in all-cause mortality (6–36%) and CVD (5–21%) at follow-up were estimated across a subsample of included studies. There was no evidence of significant interaction by age, sex, health conditions or behaviors (e.g., alcohol use, smoking status, diet) among studies that tested for interactions. Studies examining dysglycemia outcomes report inconsistent findings, partially due to heterogeneity across studies of glycemia-related biomarker outcomes, analytic approaches, and sample characteristics.

**Conclusions:**

Evidence from longitudinal data consistently demonstrated that walking an additional 1000 steps per day can help lower the risk of all-cause mortality, and CVD morbidity and mortality in adults, and that health benefits are present below 10,000 steps per day. However, the shape of the dose-response relation is not yet clear. Data are currently lacking to identify a specific minimum threshold of daily step counts needed to obtain overall health benefit.

## Background

The health benefits of physical activity for people of all ages, fitness levels, and sociodemographic backgrounds are well-documented [1–4]. Walking is a central component of physical activity and public health promotion efforts [1, 5], and daily step counts have demonstrated success as a target for achieving recommended amounts of physical activity in adults [6–8]. In addition, the expansion of wearable activity monitors and smartphones with activity-tracking capabilities onto the commercial market over the last 15 years has brought the “steps per day” activity metric into homes and healthcare systems across the world [9–11]. The increasing presence and use of self-monitoring devices and the accessibility of daily step counts as a physical activity target among the general population make it an important adjunct to current public health guidelines [7].

Despite these emerging benefits of steps for public health, recently-released guidelines for physical activity concluded there was insufficient evidence to recommend the number of daily steps needed for health. The associations of daily step counts with subsequent mortality, cardiovascular disease (CVD) risk, and type 2 diabetes were examined as part of the *2018 Physical Activity Guidelines Advisory Committee Report* [6, 7]. This review was limited, however, by the small number of studies available. The authors identified 11 total articles for review, of which only 7 were longitudinal design [6, 7]. This area of study is rapidly evolving, and a number of longitudinal studies (many with large sample sizes) have since been published. To inform future public health guidelines for physical activity, it will be important to summarize the evidence for the prospective relationship between device-measured daily step counts and health outcomes. The present review extends the previous analysis and provides an updated description of the association between daily step counts and subsequent CVD morbidity or mortality, dysglycemia, and all-cause mortality in adults and the patterns of these associations. We also investigate if these associations vary by age, sex, or moderating variables (e.g., weight status, alcohol use). The findings from this review will help form the evidence base for the number of steps per day needed for health benefit.

## Methods

### Search strategy

A systematic literature search was conducted in Medline, Embase, CINAHL, and the Cochrane Library from inception to August 1, 2019. The search strategy combined terms related to daily step count measured by pedometer or accelerometer with terms related to mortality, CVD, and dysglycemia (including type 2 diabetes and biomarkers such as insulin, blood glucose**,** HOMA, and HbA1c)**.** A search filter limited results to randomized controlled trials and cohort studies. Articles addressing congenital heart disease were excluded.

The same strategy was used to search Medline and Embase on the OVID platform and adapted for CINAHL and Cochrane search engines. The search terms used for each database are provided in Supplemental materials. Additional citations were identified by expert consultation and review of secondary sources. The systematic review search strategy was registered with PROSPERO (CRD42020142656).

### Study selection

To be included in the review, studies had to (1) use a device-based measure of daily step counts; (2) report on the association between daily step counts and mortality, CVD incidence (coronary heart disease/ischemic heart disease, coronary artery disease, stroke, heart failure, and/or metabolic syndrome), or type 2 diabetes (incidence of type 2 diabetes, dysglycemia, or changes in measures of insulin, blood glucose, HOMA, and/or HbA1c) in a prospective design; (3) be written in English; and (4) include only adults (≥18 years of age). Studies conducted in diseased populations (e.g., heart failure, hospitalized patients, hepatitis, end-stage renal disease) were not included in this review. Study selection was performed independently by two researchers (KH and EH), and differences relating to inclusion and exclusion criteria were resolved by consensus of the authors.

**Fig. 1:**
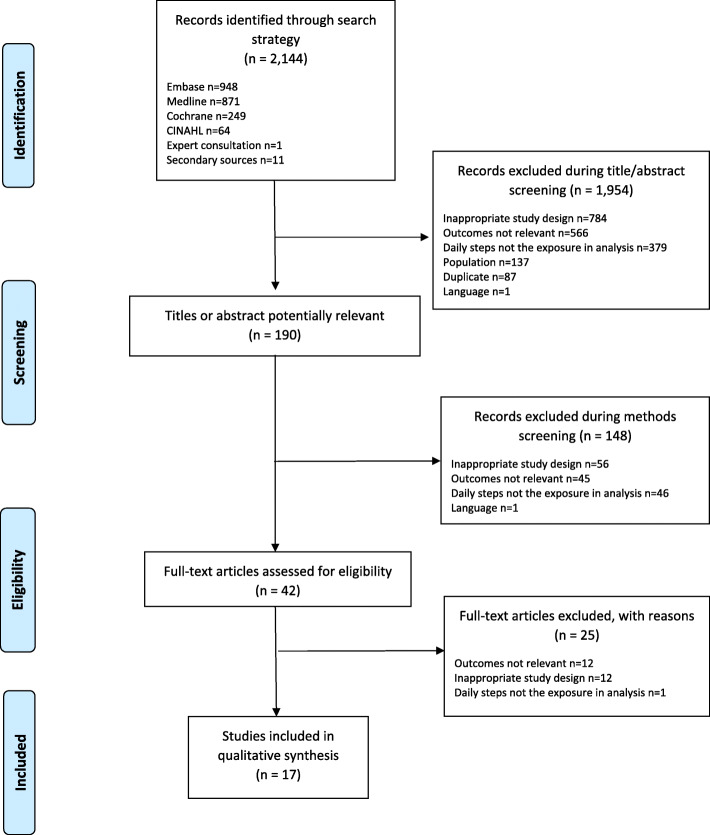
Article screening process

Titles and abstracts of 2144 citations were independently reviewed against inclusion criteria by EH and KH. Authors excluded 1954 articles at this stage. Review of the study methods of the remaining 190 articles resulted in further exclusions. The full text of 42 articles deemed potentially relevant were retrieved for further independent review. Among those, 25 were excluded, leaving 17 studies included in the analysis. The accompanying chart (Fig. 1) details the results of the screening process.

### Data extraction

Two authors (KH and EH) extracted data from the 17 included studies into a preformatted table adapted for this review [12]. Descriptive information included primary outcomes; covariates included in the statistical models; sample size; characteristics of study participants — including age, sex, and other clinical characteristics; activity levels of the sample at baseline; exposure measurement — including duration of monitoring period, type of device, and number of valid days required for inclusion (accelerometer studies only); statistical methods used; outcome follow-up time; and authors and year of publication. The rationale for this study was to describe and discuss the patterns of the associations between daily step counts and health outcomes, and as such, data are synthesized and presented narratively. To aid interpretation and comparison across studies, reporting of the exposure of daily step counts was standardized to 1000 steps per day difference at the baseline assessment for each health outcome. We chose the 1000 step per day increment because it required limited data manipulation to achieve harmonization across studies, as 7 out of 10 of the harmonized studies reported 1000 steps/day increments. For those studies that did not use 1000 steps per day as the base unit of analysis, we calculated standardized risk reduction scores per 1000 steps per day. For example, if the outcome measure reported was a hazard ratio with a unit of analysis was 2000 steps per day, the standardized risk reduction score was calculated as the square root of the hazard ratio and then converted to a percent risk reduction.

### Assessment of study quality

KH and EH developed a quality assessment tool based on the STROBE (Strengthening the Reporting of Observational studies in Epidemiology) Statement [13] and quality assessment tool developed by Fuzeki and colleagues [14]. For each study, information on study quality was extracted by two authors (KH and EH); differences in this assessment were discussed until consensus was reached. Study quality was determined by answers to the questions listed in Supplementary Table 1**.** Briefly, study quality criteria included assessment of study purpose, participant selection, measurement, reporting, statistical methods, and study limitations. Items were coded as ‘yes/present’ (1) or ‘no/unclear/not reported’ (0). Studies scoring 8–11 were classified as high quality, those scoring 5–7 points were classified as moderate quality, and those scoring below 5 points were classified as low quality (max score = 11) [14].

## Results

### Characteristics of the included studies

Seventeen studies from 13 different cohorts were included in the systematic review; five studies assessed all-cause mortality [15–19], four assessed cardiovascular events [20–23], and eight assessed dysglycemia [24–31]. Follow-up measurements of health outcomes ranged from three months [25] to 10 years [15, 19]. Sample sizes ranged from 47 [28] to 16,741 [18], with samples comprised of 46.9% female participants on average. Mean age ranged from 49.7 [27] to 78.9 [20] years; the average baseline median number of daily step counts across studies was approximately 6000 (range 2681 [20] to 10,969 [24]). The studies were geographically diverse with participants from over 40 countries; these included Australia, the United States, the United Kingdom, South Africa, China, and Japan. Two studies of Australian adults in the AusDiab cohort reported nearly twice the number of daily steps at baseline compared to other samples (approximately 10,600 compared to approximately 5500), which may be partially due to population-level differences in physical activity behavior across countries. A full description of the included studies can be found in Table 1.

**Table 1:** Description of included prospective studies of steps per day, by outcome category

Outcome category (number of studies)	Reference	Cohort or Study	Cohort characteristics (country)	Outcome measure(s)	Outcome follow-up time (years)	Sample characteristics				
						Sample size (events; %)	Female (%)	Age, years Mean ± SD or Median (IQR)	BMI, kg/m^2^ Mean ± SD or Median (IQR)	Baseline daily step counts Mean ± SD or Median (IQR)
**All-cause mortality** **(*N*** **= 5)**	Dwyer, 2015 [15]	Tasped cohort (3 pooled population cohorts [AusDiab, TASOAC, TASCOG])	AusDiab: 1136 adults aged ≥25 years in 1999–2000 (Aus) TASOAC: 1041 adults ages 50–80 years in 2002–2004 (Aus) TASCOG: 399 adults ages 60–86 years in 2005 (Aus)	All-cause mortality	10	*n* = 2576 (219; 8.5%)	52.4	58.8 ± 13.2	27.4 ± 4.8	8856 ± 4510
	Fox, 2015 [16]	Project OPAL	240 adults aged ≥70 years (UK)	All-cause mortality	4–5	*n* = 201 (33; 16.4%)	48.8	Age, years (%) 70–74.9: 36.6 75–79.9: 26.8 80–84.9: 24.9 85+: 11.7	BMI, kg/m^2^(%) < 25.0: 34.3 25.0–29.9: 37.6 ≥30.0: 28.2	Tertiles, (%) < 3196: 31.8 3196–5170: 33.3 > 5170: 34.8
	Jefferis, 2019 [17]	British Regional Heart Study	7735 men aged 40–59 recruited in 1978–1980 (UK)	All-cause mortality	5	*n* = 1181 (194; 16.4%)	0	78.4 ± 4.6	27.1 ± 3.8	4938 ± 2794
	Lee, 2019 [18]	Women’s Health Study	39,876 US women aged ≥45 recruited in 1992–2004	All-cause mortality	4	*n* = 16,741 (504; 3%)	100	72.0 ± 5.7	26.2 ± 5.0	5499 (SD not reported)
	Yamamoto, 2018 [19]	N/A	600 adults aged 70 years in 1998 (Japan)	All-cause mortality	10	*n* = 419 (76; 18.1%)	45.6	71.0 ± 0.0	22.6 ± 2.9	6470 ± 2732
**CVD (*N*** **= 4)**	Cochrane, 2017 [20]	LIFE RCT	1600 mobility-limited older adults aged 70–89 years in 2010–2013 (US)	Composite of MI, silent MI, hospitalized angina, congestive heart failure, revascularization with bypass surgery or percutaneous angioplasty, aortic aneurysm, peripheral artery disease, stroke, or transient ischemic attack	0.5, 1, and 2	*n* = 1590 (234; 14.7%)	67.2	78.9 ± 5.2	30.1 ± 5.9	2681 ± 1475
	Huffman, 2014 [21]	NAVIGATOR trial	9306 adults with impaired glucose tolerance and existing CVD (if ≥50 years) or with at least 1 additional CVD risk factor (if ≥55 years) in 2002–2004 (multiple)	Cardio-metabolic risk score	6	*n* = 7118 (N/A)	50.6	63.0 (58.0–69.0)	29.6 (26.7–33.2)	6178 ± 3833
	Jefferis, 2019 [22]	British Regional Heart Study	7735 men aged 40–59 recruited in 1978–1980 (UK)	MI, stroke, or heart failure morbidity or mortality	5	*N* = 1181 (122; 10.3%)	0	78.4 ± 4.6	27.1 ± 3.8	4938 ± 2794
	Yates, 2014 [23]	NAVIGATOR trial	9306 adults with impaired glucose tolerance and existing CVD (if ≥50 years) or with at least 1 additional CVD risk factor (if ≥55 years) in 2002–2004 (multiple)	Composite of time to death from cardiovascular causes, non-fatal MI, or non-fatal stroke	5	*n* = 9018 (531; 5.9%)	51.0	63.0 (58.0–69.0)	29.6 (26.8–33.3)	6245 (4065–9157)
**Dysglycemia (*N*** **= 8)**	Kraus, 2018 [26]	NAVIGATOR trial	9306 adults with impaired glucose tolerance and existing CVD (if ≥50 years) or with at least 1 additional CVD risk factor (if ≥55 years) in 2002–2004 (multiple)	Diabetes	5	*n* = 9306 (3254; 35.0%)	50.6	65.0 (59.0–71.0)	31.2 (27.7–35.4)	6205 ± 3727
	Ponsonby, 2011 [27]	AusDiab	1136 adults aged ≥25 years in 1999–2000 (Aus)	Incident dysglycemia	5	*n* = 458 (26; 5.7%)	55.9	49.7 ± 1.5	26.1 ± 0.4	10,733 (7695–13,833)
	Dwyer, 2011 [24]	AusDiab	1136 adults aged ≥25 years in 1999–2000 (Aus)	Insulin sensitivity	5	*n* = 592 (N/A)	54.9	50.8 ± 12.3	26.5 ± 3.9	Men: 10172 (7435–13,928) Women: 10969 (7889–14,402)
	Herzig, 2014 [25]	PreDiabEx RCT	78 adults at high risk for type 2 diabetes (Finland)	Insulin sensitivity, fasting glucose	0.25	*n* = 68 (N/A)	73.5	58.8 ± 10.3	31.7 ± 5.3	5130 ± 3424
	Siddiqui, 2018 [31]	N/A	95 adults aged 18–65 with type 2 diabetes (South Africa)	HbA1c	0.25	*n* = 95 (N/A)	67.4	54.7 ± 7.1	33.2 ± 6.4	3811 ± 1683
	Tudor-Locke, 2004 [28]	First Step Program RCT	146 adults with diabetes ages 40–60 years (year not given) (Canada)	Fasting glucose, fasting insulin, HbA1c	0.3 and 0.5	*n* = 47 (N/A)	44.7	52.7 ± 5.2	33.3 ± 5.6	6011 ± 2496
	Van Dyck, 2013 [29]	N/A	92 adults with type 2 diabetes ages 35–75 in 2007	HbA1c, fasting glucose	0.5 and 1	*n* = 92 (N/A)	31.0	62.0 ± 9.0	30.0 ± 2.8	5021 ± 2591
	Yates, 2015 [30]	NAVIGATOR trial	9306 adults with impaired glucose tolerance and existing CVD (if ≥50 years) or with at least 1 additional CVD risk factor (if ≥55 years) in 2002–2004 (multiple)	Fasting glucose	4	*n* = 3964 (N/A)	50.4	63.0 (58.0–68.0)	29.0 (26.3–32.6)	6501 (4235–9323)

Abbreviations: *SD* standard deviation; *IQR* interquartile range; *BMI* body mass index; *Aus* Australia; *N/A* not applicable; *RCT* randomized controlled trial; *MI* myocardial infarction; CVD, cardiovascular disease

The methods used to measure daily step counts in the 17 studies are described in Table 2. Eleven studies measured steps with pedometers [15, 19, 21, 23, 24, 26–31] and six with accelerometers [16–18, 20, 22, 25]. Seven studies [16–19, 21, 22, 26] measured daily step counts only at baseline; ten studies [15, 20, 23–25, 27–31] included at least one *repeated measure* of step counts and subsequent health events. All studies used devices placed at the waist or hip (data not shown). Ten studies measured steps for seven consecutive days [16–23, 26, 30], two studies measured for at least 3 months [25, 31], and five studies measured for durations less than 7 days [15, 24, 27–29]. Among studies using accelerometers, all but one [25] required at least 10 h per day of wear time, though the number of days required for study inclusion ranged from three [17, 20, 22] to five [16]. Two studies [23, 26], both from the NAVIGATOR trial, used multiple imputation to address missing pedometer data from the seven-day monitoring period. All other studies excluded respondents with insufficient daily step count data (range as percent of analytic sample: < 1–43%).

**Table 2:** Description of methods used to measure daily step counts in included studies

Outcome category (number of studies)	Reference	Pedometer or Accelerometer	Device(s)	Monitoring period	Time point of steps/day measurement	Data processing criteria
**All-cause mortality (*N*** **= 5)**	Dwyer, 2015 [15]	Pedometer	Omron HJ-003, Omron HJ-102, and Yamax Digi-Walker	2 consecutive days (including ≥1 weekday)	Baseline and mean 3.7 years (subset)	None reported
	Fox, 2015 [16]	Accelerometer	ActiGraph GT1M	7 consecutive days	Baseline	Valid wear time: ≥10 h/day and ≥ 5 days
	Jefferis, 2019 [17]	Accelerometer	ActiGraph GT3X	7 days during waking hours	Baseline	Valid wear time: ≥10 h/day and ≥ 3 days
	Lee, 2019 [18]	Accelerometer	ActiGraph GT3X	7 consecutive days during waking hours	Baseline	Valid wear time: ≥10 h/day and ≥ 4 days
	Yamamoto, 2018 [19]	Pedometer	EX-100S	7 consecutive days during waking hours	Baseline	Valid wear time: ≥3 days
**CVD (*N*** **= 4)**	Cochrane, 2017 [20]	Accelerometer	ActiGraph GT3X	7 consecutive days during waking hours	Baseline, 0.5, 1, and 2 years	Valid wear time: ≥10 h/day and ≥ 3 days for at least one time point
	Huffman, 2014 [21]	Pedometer	Accusplit AE120	7 consecutive days during waking hours	Baseline	None reported
	Jefferis, 2019 [22]	Accelerometer	ActiGraph GT3X	7 days during waking hours	Baseline	Valid wear time: ≥10 h/day and ≥ 3 days
	Yates, 2014 [23]	Pedometer	Accusplit AE120	7 consecutive days during waking hours	Baseline and 1 year	None reported
**Dysglycemia (*N*** **= 8)**	Kraus, 2018 [26]	Pedometer	Accusplit AE120	7 consecutive days during waking hours	Baseline	None reported
	Ponsonby, 2011 [27]	Pedometer	Omron HJ-003, Omron HJ-102	2 consecutive days (including ≥1 weekday)	Baseline and 5 years	None reported
	Dwyer, 2011 [24]	Pedometer	Omron HJ-003, Omron HJ-102	2 consecutive days	Baseline and 5 years	None reported
	Herzig, 2014 [25]	Accelerometer	Newtest Exercise Monitor	Every day for 3 months during waking hours	Duration of study	Valid day: > 1000 steps
	Siddiqui, 2018 [31]	Pedometer	Not reported	Every day for 4 months during waking hours	Duration of study	None reported
	Tudor-Locke, 2004 [28]	Pedometer	Yamax SW-200	3 consecutive days (including 1 weekend day) during waking hours	Baseline, 0.3, and 0.5 years	None reported
	Van Dyck, 2013 [29]	Pedometer	Yamax DigiWalker SW200	5 consecutive days (including ≥1 weekend day) during waking hours	Baseline, 0.5, and 1 year	None reported
	Yates, 2015 [30]	Pedometer	Accusplit AE120	7 consecutive days	Baseline and 1 year	Valid wear time: ≥1 day

The analytic methods from all included studies are described in Supplementary Table 2. The covariate measures in each study varied considerably; however, age, sex, BMI and/or waist circumference, and smoking status were the most commonly included covariate measures across all studies (Supplementary Table 2). Six studies reported sensitivity analyses excluding the first 1–3 year(s) of follow-up [15, 17–19, 22, 23]. Five studies assessed for significant differences in the association between daily step counts and outcomes by selected clinical, behavioral, and/or demographic characteristics [15, 20, 23, 24, 26]. Thirteen studies [15–24, 26, 27, 31] analyzed total daily step counts as a continuous variable; seven analyzed daily steps across quantiles [15–19, 22, 25]. Supplementary Table 3 shows the quality criteria scores of each study. Ten studies were of high quality [16–19, 21–24, 26, 30] and seven studies were of moderate quality [15, 20, 25, 27–29, 31].

### Daily step counts and all-cause mortality

**Fig. 2:**
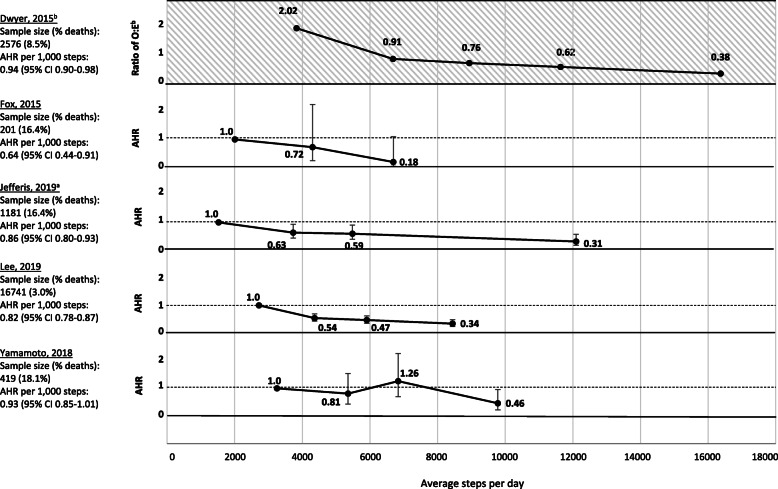
Associations of quantile medians^a^ of baseline daily step counts and all-cause mortality across included studies. Abbreviations: O:E, ratio of observed and expected deaths, AHR, adjusted hazard ratio; CI, confidence interval; Note: Error bars represent the lower and upper bounds of the 95% confidence interval. ^a^Quantile medians of daily step counts at baseline were not reported by Jefferis and colleagues [17]. Therefore, the midpoints of the quantile ranges of daily step counts at baseline were plotted instead. ^b^Dwyer, 2015 reported the distribution of obeserved and expected deaths (confidence intervals not reported) by quantiles of daily step counts at baseline. Shading on the figure indicates the distinction in measure of association from the other four studies, all of which assessed the associations of quantiles of daily step counts at baseline and the adjusted hazard ratio for all-cause mortality

Among the five studies assessing all-cause mortality, three studies of high quality [16, 18, 19] and two studies [15, 17] of moderate quality reported significant associations between greater daily step counts at baseline (continuous) and less risk of all-cause mortality. All but one study [19] reported significant evidence of a linear relationship between steps per day and all-cause mortality risk (Table 3). Follow-up time across studies ranged from 4 years [16, 18] to 10 years [15, 19], and sample sizes ranged from 201 [16] to 16,741 [18] participants. In addition to analyzing daily step counts as a continuous variable, all five studies analyzed daily step counts by quantiles (Fig. 2). Each study reported significantly less risk of all-cause mortality among those in the greatest step count group compared to those in the least step count group. The average daily step counts for each quantile in Fig. 2 was plotted using quantile medians. One study compared the observed-to-expected mortality ratio across quantiles of average daily step counts [15], and four used adjusted hazard regression to compare risk of all-cause mortality in the lowest quantile of average daily step counts to all other quantiles [16–19].

**Table 3:** Association of daily step counts at baseline and outcome in selected studies^a^

Reference	Sample size (events; %)	Age, years mean ± SD or median (IQR)	Outcome	Follow-up time (years)	Study reported			Standardized^b^
					Daily step count unit	Tested for non-linearity	Risk reduction	Risk reduction
Dwyer, 2015 [15]	2576 (219; 8.5%)	58.8 ± 13.2	All-cause mortality	10	1000 steps/day	Yes	6%	6%
Yamamoto, 2018^c^ [19]	419 (76; 18.1%)	71.0 ± 0.0	All-cause mortality	10	1000 steps/day	Yes	7%^c^	7%^c^
Jefferis, 2019 [17]	1181 (194; 16.4%)	78.4 ± 4.6	All-cause mortality	5	1000 steps/day	Yes	14%	14%
Fox, 2015 [16]	201 (33; 16.4%)	Not reported	All-cause mortality	4–5	1000 steps/day	No	36%	36%
Lee, 2019 [18]	16,741 (504; 3%)	72.0 ± 5.7	All-cause mortality	4	1000 steps/day	Yes	18%	18%
Jefferis, 2019 [22]	1181 (122; 10.3%)	78.4 ± 4.6	Myocardial infarction, stroke, or heart failure morbidity or mortality	5	1000 steps/day	Yes	14%	14%
Yates, 2014 [23]	9018 (531; 5.9%)	63.0 (58.0–69.0)	Composite of time to death from cardiovascular causes, non-fatal myocardial infarction, or non-fatal stroke	5	2000 steps/day	Yes	10%	5%
Cochrane, 2017 [20]	1590 (234; 14.7%)	78.9 ± 5.2	Composite of cardiovascular disease events	2	500 steps/day	No	11%	21%
Kraus, 2018 [26]	9306 (3254;35.0%)	65.0 (59.0–71.0)	Diabetes incidence	5	2000 steps/day	Yes	4%	2%
Ponsonby, 2011 [27]	458 (26; 5.7%)	49.7 ± 1.5	Incident dysglycemia	5	1000 steps/day	Yes	13%	13%

Abbreviations: *SD* standard deviation; *IQR* interquartile range

^a^7 studies not included (Huffman, 2014; Dwyer, 2011; Herzig, 2014; Siddiqui, 2019; Tudor-Locke, 2004; Van Dyck, 2013; Yates, 2015) due to analytic methods that could not be harmonized

^b^Exposure of daily steps standardized to 1000 steps/day at baseline and an assumed linear association

^c^Yamamoto, 2018 reported that the linear association between daily step counts and all-cause mortality was not statistically significant. All other studies in the table reported finding a statistically significant linear association between daily step counts and the outcome

Table 3 summarizes the findings of all-cause mortality studies. Based on an assumed linear association between daily step counts and mortality, for each study, reporting of the exposure of daily step counts was standardized to 1000 steps per day difference at the baseline assessment. The estimates for mortality are based on 21,118 participants and 103,723 person-years. The standardized risk reduction across the five studies per each 1000 daily steps increase at baseline ranged from 6% [15] to 36% [16].

One study explored prospective *change* in daily step counts and subsequent all-cause mortality risk among a subsample of participants [15]. The authors reported that any increase in daily step count over time, compared to no change or decrease over time, was associated with reduced mortality (AHR, 0.38, 95% CI, 0.21–0.70, P = 0.002). This finding was difficult to interpret, however, because the reference group included those with either no change or reduced step counts. In addition, the authors noted more deaths were observed than expected in the reference group. Four studies [15, 17–19] conducted sensitivity analyses by removing deaths in the first 1–3 years of follow-up and reported no meaningful differences; of studies that tested for interactions, no significant interaction effects between steps and sex [15, 19], age [15, 17], or health conditions (e.g., weight status, chronic disease) and behaviors (e.g., alcohol use, diet) were reported (data not shown) [15, 17, 18].

### Daily step counts and CVD morbidity or mortality

Among the four studies assessing CVD morbidity or mortality, three studies of high quality [21–23] and one study of moderate quality [20] reported significant associations between greater daily step counts at baseline (continuous) and lower risk of CVD. These prospective studies reported CVD outcomes including calculated cardio-metabolic risk score [22], composite CVD morbidity or mortality — which included myocardial infarction, stroke, or heart failure [23], and two different composites of CVD incidence and mortality [21, 24]. The follow-up time across all four studies ranged from 6 months [21] to 6 years [22]. Sample size ranged from 1181 (a prospective cohort of men) [22] to 9018 [24] participants.

All four studies reported significant evidence of a linear relationship between greater steps per day and lower risk of CVD morbidity or mortality. The reported risk reduction in CVD varied based on unit of difference in daily step counts. One study assessed quartiles of baseline daily step counts and reported significantly lower risk of CVD among those in the greater daily step count groups compared to those in the least step counts group [22]. Table 3 summarizes the findings of three studies [20, 22, 23] whose analytic methods and results were comparable. The other study identified in this review was not included in this table because the health outcome (composite cardio-metabolic risk score) was not consistent with the other studies [21]. Based on an assumed linear association between daily step counts and CVD morbidity and mortality, for each study, reporting of the exposure of daily step counts was standardized to 1000 steps per day difference at the baseline assessment. The estimates for CVD are based on 11,789 participants and 54,175 person-years. The standardized risk reduction in CVD events across three studies per each 1000 daily steps increase at baseline ranged from 5% [23] to 21% [20].

One study explored change in daily steps and subsequent CVD across repeated measurement periods [23]. Two studies conducted sensitivity analyses of previous CVD history and reported it had no effect on the association between daily step counts and incident CVD [20, 23]. Only one study conducted interaction analyses (Supplementary Table 2), and found no significant effects of age, sex, or baseline daily step counts [23].

### Daily step counts and dysglycemia

Eight prospective studies [24–30] examined the relationship between daily step counts and dysglycemia; three studies were high quality [24, 26, 30] and five were moderate quality [25, 27–29, 31]. Four studies examined associations in the context of a lifestyle intervention for adults with, or at high risk of type 2 diabetes mellitus [25, 28, 29, 31]; two were conducted in the AusDiab prospective cohort [24, 27]; and two reported on the NAVIGATOR trial [26, 30]. The four lifestyle intervention studies [25, 28, 29, 31] were small (< 100 participants) and had short follow-up times (≤1 year) compared to the AusDiab and NAVIGATOR studies. Three of the intervention trials targeted increased walking or daily steps [28, 29, 31], and one targeted increased physical activity [25]. All four lifestyle intervention studies were treated as cohorts and the associations between daily step counts and the outcomes were assessed across the whole sample, regardless of study arm. These prospective studies reported on a number of dysglycemia outcomes including blood glucose levels and HbA1c, insulin resistance, 2-h glucose, insulin sensitivity (e.g., HOMA-IR), and incident dysglycemia or type 2 diabetes.

Results across the longitudinal studies were mixed. Two studies examined the association between daily step counts and insulin sensitivity (assessed by HOMA-IR): one small study reported no association [25] and one large study reported a weak inverse association [24]. Six studies examined the association between daily step counts and fasting glucose or HbA1c: four reported no significant associations [25, 29–31], and two reported significant inverse associations [27, 28]. Two small studies with short follow-up examined daily step counts and fasting insulin; both reported no association [25, 28]. Three studies examined the association between daily step counts and 2-h glucose: one reported no association [25] and two reported weak inverse associations [28, 30]. Two larger studies with 5-year follow-up examined the association between daily step counts and incident dysglycemia or type 2 diabetes and found significantly reduced risk with greater daily step counts [26, 27]. Four studies tested interactions with confounding variables [24, 26, 27, 30] (Supplementary Table 2); they reported no significant effects of sex [24, 26, 27], age [27], or health measures [24, 27, 30] (e.g., insulin sensitivity, smoking status, glucose tolerance, cardiovascular conditions).

Table 3 summarizes the findings of two studies [26, 27] whose analytic methods and results were comparable. The six other studies [24, 25, 28–31] identified in this review were not included in this table because no point estimate for risk reduction was reported for dysglycemia outcomes. Four studies tested linear models and all reported significant evidence of linearity for the relationship between daily step counts and dysglycemia outcomes [24, 26, 27, 30]. Based on an assumed linear association between daily step counts and dysglycemia, for each study, reporting of the exposure of daily step counts was standardized to 1000 steps per day difference at the baseline assessment. Two studies had standardized risk reduction estimates for dysglycemia outcomes: 2% [26] for incident diabetes among individuals with impaired glucose tolerance, and 13% [27] for incident dysglycemia among individuals with normal glucose tolerance.

## Discussion

This systematic review provides evidence of the benefit of increasing steps per day for health: taking more steps per day was associated with lower risk of all-cause mortality, and lower risk of CVD morbidity or mortality. Even at low levels of activity, taking an additional 1000 steps per day was associated with lower risk of all-cause mortality, and lower risk of CVD morbidity or mortality. These associations appear to hold across age, gender, and weight status.

This systematic review of 17 prospective studies extends the findings of the *2018 Physical Activity Guidelines Advisory Committee Report*, which was limited to seven prospective studies, and provides a current summary of the prospective association between daily steps and mortality and cardiometabolic biomarkers [6, 7]. Our results are in agreement with previous studies which have reported that increases in walking (primarily based on self-report) are associated with lower risk of all-cause mortality, and CVD mortality and risk factors in adults [3, 32, 33].

We identified three new longitudinal studies of daily steps and all-cause mortality, all with large numbers, that were not included in the previous review [6]. For each 1000 step per day increase at baseline, we report a range of possible risk reductions in all-cause mortality (6–36%) over 4–10 years. This is a considerable expansion from the 6–7% risk reduction reported in a previous review with fewer studies [6]. Importantly, reduced risk of mortality was observed even at low levels of daily steps, below the commonly ascribed 10,000 steps per day threshold (Fig. 2). There were too few studies to test the shape of the dose-response relationship, though our results suggest less mortality risk in adults with the greatest compared to the least daily step counts, a suggestion of “more is better” with respect to mortality risk. We identified two new longitudinal studies of daily steps and CVD morbidity or mortality that were not included in a previous review [6]. For each 1000 step per day increase at baseline, we report a range of possible risk reductions in CVD morbidity and mortality (5–21%) over 2–5 years. This is a considerable expansion from the 5% risk reduction reported in a previous review with fewer studies [6].

In contrast to our findings for all-cause mortality and CVD mortality and morbidity, our study could not definitively characterize the association between dysglycemia and diabetes using the eight studies identified. These inconsistent findings may be because of the heterogeneity of glycemia-related biomarker outcomes, analytic approaches, and sample characteristics. However, results from four large cohort studies with longer follow-up time do suggest a beneficial effect of increasing daily steps on incident diabetes (in adults with and without impaired glucose tolerance), fasting glucose and insulin sensitivity (in adults without impaired glucose tolerance), and 2-h glucose (in adults with impaired glucose tolerance) [24, 26, 27, 30]. The findings from these four cohort studies are consistent with the findings of other systematic reviews that find that diabetes risk declines with increased levels of overall physical activity [34, 35]. The *2018 Physical Activity Guidelines Advisory Committee* also concluded that there was limited evidence of an association between daily step counts and reduced risk of type 2 diabetes incidence [7, 26]; and we did not identify any new data in the present report. Additional evidence from longitudinal studies is needed to determine the association between daily steps and dysglycemia outcomes and to provide dose-response data.

Our findings appear to be robust to threats from confounding or bias. For all health outcomes, the effect of daily step counts was robust to adjustment for sociodemographic (e.g., age, sex), individual (e.g., weight status, disease history), and lifestyle characteristics (e.g., smoking, alcohol use). The generalizability of these conclusions is also supported by the representation of men and women, healthy and at-risk populations, and diverse geographical areas in this systematic review. However, only five studies (representing just two unique prospective studies) reported the racial/ethnic characteristics of the sample [20, 21, 23, 26, 30] and just three studies reported the socioeconomic (e.g., education, social class, income) characteristics [16, 20, 22]. Based on the available information, it appears the majority of participants in the studies included in this review were white adults, from developed countries, and of higher socioeconomic status. As a result, it is not clear how generalizable our results are to racial/ethnic minorities or low- and middle-income persons or settings.

Several methodological issues in the individual studies could have affected the findings in our review. First, we noted large disparities in the number of participants included in the analyses of daily steps counts and health outcomes compared to 1) the overall cohort sample sizes and 2) the number of adults with steps data at baseline. The issue of missing follow-up data among those participants with baseline exposure data also went largely unaddressed in these prospective studies (only 18% reported methods for dealing with missing data; Supplementary Table 3, item #6). Both of these issues could have biased associations or limited the generalizability of study results. Second, although the studies included in the review were all moderate-to-high quality, there was considerable heterogeneity in sample size and outcome follow-up time. Only eight studies, representing four unique prospective cohorts, reported on sample sizes ≥1000 participants and follow-up time period ≥4 years; the remaining studies were substantially smaller and/or had follow-up time of only 3 months to 2 years. This field would benefit from larger studies of longer duration (> 5 years) that provide robust estimates of the association of daily step counts with health outcomes. Finally, the cardiovascular and dysglycemia outcome measures also varied considerably across studies, with several using composite measures of differing cardiovascular events and dysglycemia markers ranging from insulin sensitivity to incident diabetes; this further complicated comparisons across studies.

We also identified methodologic limitations specifically pertaining to exposure assessment of steps in the individual studies that warrant further discussion in the context of future research. First, we noted that different wearable devices were used to assess steps. Pedometers, while extremely useful for epidemiologic assessment, often lack the ability to store data in memory on the device, requiring participants to complete step logs [36]. Newer devices, such as accelerometers, have demonstrated excellent reliability and validity for walking, though this depends on where they are placed (usually waist-worn) and the model of the accelerometer [36, 37]. These devices often rely on proprietary algorithms to estimate step counts, making data interpretation challenging. The increasing popularity of wrist-worn consumer devices and smartphones for monitoring step counts [38] is sure to add another element of measurement variability and may introduce issues when translating research findings into public health recommendations. Second, although recommended study protocols for length and duration of accelerometer wear exist, our review found that over one-third did not carry out exposure measurement using these conventional methods [39] (Supplementary Table 3, item #4). These protocols include ≥4 days of monitoring (pedometer and accelerometer studies), valid wear time defined as ≥10 h/day (accelerometer studies only); reporting data processing criteria (accelerometer studies only). Despite these inconsistencies, we report that as little as 2–7 days of assessment across studies was predictive of mortality and CVD outcomes. Standardized methods across studies will improve comparability, and future efforts to ensure adequate measurement and analysis of step count data will greatly improve this field of research. As activity monitoring devices are being integrated successfully into numerous large-scale prospective trials, repeated measures of step count data and subsequent health events will be available. The logistical and analytic insights from these studies could improve standardization as well as our understanding of the prospective relationship [40–42].

Our systematic review examined studies published from over the past 70 years. Despite a robust search strategy, we identified only a small number of longitudinal studies assessing daily steps and health outcomes. Many of these studies were conducted recently (past 1–8 years), demonstrating this as a new area of research. In addition, while 10 of the included studies included at least one repeated measure of daily step counts, only two studies prospectively examined the association of change in daily step counts and subsequent risk of the outcome [15, 23]. However, a recent study found that stability in accelerometer measured daily step counts appears stable over 2–3 years [41]. Although originally planned, we were not able to conduct meta-analyses of these studies due to the lack of standardized metrics (e.g., per 1000 steps, quantiles) and heterogeneity of study designs and populations. The lack of detailed information (e.g., point estimate and standard error) in the papers also precluded our ability to harmonize across the studies. To address these needs, we urge authors to provide detailed information in future publications on risk factor analyses and related parameters required for meta-analysis [43–45].

This is the largest systematic review to date of prospective associations between daily step counts and important health outcomes, reporting on 17 geographically diverse studies drawn from 12 unique cohorts. The *2018 Physical Activity Guidelines Advisory Committee* identified a need for more evidence of the longitudinal associations between daily steps and risk of mortality, CVD, and dysglycemia. This systematic review addresses this need and identifies opportunities for additional studies to advance the field and build the evidence base around daily steps for health. Information on steps and health may be used to augment current recommendations on the amount of physical activity needed for health. Although physical activity (e.g., frequency, intensity, duration) may be easy to intuit for someone who exercises regularly, individuals who participate in activities such as gardening, house cleaning, or walking for errands may have difficulty determining the duration and intensity of these activities. Among such people, using daily steps obtained from activity trackers may be another way to promote public health guidelines and help individuals achieve the recommended amounts of physical activity. Another strength of the current review is that it investigates individual cardiometabolic biomarkers (e.g., insulin sensitivity, fasting glucose, cardio-metabolic risk score) in addition to global measures of cardiovascular and metabolic health (e.g., type 2 diabetes, CVD).

In conclusion, there was consistent evidence from longitudinal data that walking an additional 1000 steps per day can help lower the risk of all-cause mortality, and CVD morbidity and mortality in adults, and that health benefits are present below 10,000 steps per day. Our review also demonstrates a current lack of data on the relationship between step counts and subsequent health outcomes to adequately inform a daily step count guideline [7, 8]. Additional evidence can come from completing new analyses of existing studies with the requisite exposure and outcome measures reported here, or by designing new studies that address some of the limitations noted in our review. These studies could include using standard methodologies and examine the relationship in different subgroups of the population. This additional evidence will help guide meaningful volume targets that can be used for health care, education, and behavioral interventions, and potentially inform the development of public health guidelines for steps and health.

## Supplementary information

**Additional file 1.**

## Data Availability

Data sharing is not applicable to this article as no datasets were generated or analyzed during the current study.

## Abbreviations

CVD: Cardiovascular disease
BMI: Body mass index
AHR: Adjusted hazard ratio
