# Supplementary material for: Systematic review of the prospective association of daily step counts with risk of mortality, cardiovascular disease, and dysglycemia
Source: Int J Behav Nutr Phys Act. 2020 Jun 20;17:78. doi: 10.1186/s12966-020-00978-9 (PMC7305604; doi:10.1186/s12966-020-00978-9)
Supplement: Supplementary file 1 — Additional file 1. [file 12966_2020_978_MOESM1_ESM.docx]

**Supplementary Materials**

**Search Terms**

**Database(s):** Ovid MEDLINE(R) and Epub Ahead of Print, In-Process & Other Non-Indexed Citations and Daily

**Dates:** 1946 to August 01, 2019
Search Strategy:

| **#** | **Searches** | **Results** |
| --- | --- | --- |
| 1 | (step adj3 count*).tw. | 1854 |
| 2 | pedometer*.tw. | 2467 |
| 3 | acceleromet*.tw. | 14388 |
| 4 | accelerometry/ | 4459 |
| 5 | actigraphy/ | 3215 |
| 6 | actigraph*.tw. | 5717 |
| 7 | or/1-6 | 23198 |
| 8 | exp Mortality/ | 362507 |
| 9 | mortality.mp. | 1075456 |
| 10 | 8 or 9 | 1185673 |
| 11 | 7 and 10 | 497 |
| 12 | exp Cardiovascular Diseases/ | 2289787 |
| 13 | cardiovascular disease*.mp. | 244225 |
| 14 | coronary heart disease*.mp. | 48989 |
| 15 | ischemic heart disease.mp. | 24951 |
| 16 | coronary artery disease.mp. | 115068 |
| 17 | exp Stroke/ | 123998 |
| 18 | stroke.mp. | 271976 |
| 19 | heart failure.mp. | 193782 |
| 20 | or/12-19 | 2486168 |
| 21 | 7 and 20 | 1903 |
| 22 | 21 not 11 | 1723 |
| 23 | 22 not (congenital heart defects/ or congenital heart disease.mp.) | 1698 |
| 24 | Diabetes Mellitus, Type 2/ | 123993 |
| 25 | type 2 diabetes.tw. | 114203 |
| 26 | insulin/ | 180517 |
| 27 | insulin.mp. | 399942 |
| 28 | Blood Glucose/ | 158292 |
| 29 | blood glucose.tw. | 65410 |
| 30 | homeostatic model assessment.mp. | 2592 |
| 31 | homa.mp. | 14898 |
| 32 | Glycated Hemoglobin A/ | 32906 |
| 33 | hba1c.tw. | 30303 |
| 34 | hemoglobin a1c.tw. | 9358 |
| 35 | or/24-34 | 591166 |
| 36 | 7 and 35 | 968 |
| 37 | 36 not metabolic syndrome.mp. | 872 |
| 38 | 37 not (11 or 23) | 632 |
| 39 | cohort studies/ | 243501 |
| 40 | longitudinal studies/ | 125291 |
| 41 | follow-up studies/ | 618619 |
| 42 | prospective studies/ | 509060 |
| 43 | retrospective studies/ | 761382 |
| 44 | (cohort or longitudinal or prospective or retrospective).tw. | 1445325 |
| 45 | (follow* adj2 up).tw. | 966168 |
| 46 | 39 or 40 or 41 or 42 or 43 or 44 or 45 | 2872937 |
| 47 | 11 and 46 | 223 |
| 48 | 23 and 46 | 477 |
| 49 | 38 and 46 | 182 |
| 50 | (Light physical activity determined by a motion sensor decreases insulin resistance, improves lipid homeostasis and reduces visceral fat in high-risk subjects: PreDiabEx study RCT).m_titl. | 1 |
| 51 | (Relationship between baseline physical activity assessed by pedometer count and new-onset diabetes in the NAVIGATOR trial).m_titl. | 1 |
| 52 | 7 and metabolic syndrome.mp. | 219 |
| 53 | 46 and 52 | 51 |
| 54 | 53 not (47 or 48 or 49) | 35 |
| 55 | limit 54 to english language | 34 |
| 56 | remove duplicates from 55 | 34 |
| 57 | (20181* or 2019*).ez. | 881046 |
| 58 | 47 and 57 | 18 |
| 59 | 48 and 57 | 33 |
| 60 | 49 and 57 | 12 |
| 61 | 54 and 57 | 6 |

**Database(s):** Embase

**Dates:** 1974 to August 01, 2019
Search Strategy:

| **#** | **Searches** | **Results** |
| --- | --- | --- |
| 1 | (step adj3 count*).tw. | 2579 |
| 2 | pedometer*.tw. | 3385 |
| 3 | acceleromet*.tw. | 18328 |
| 4 | accelerometry/ | 6302 |
| 5 | actigraphy/ | 7520 |
| 6 | actigraph*.tw. | 9914 |
| 7 | or/1-6 | 32431 |
| 8 | exp Mortality/ | 1002550 |
| 9 | mortality.mp. | 1364695 |
| 10 | 8 or 9 | 1365628 |
| 11 | 7 and 10 | 914 |
| 12 | exp Cardiovascular Diseases/ | 3846018 |
| 13 | cardiovascular disease*.mp. | 372821 |
| 14 | coronary heart disease*.mp. | 67996 |
| 15 | ischemic heart disease.mp. | 137998 |
| 16 | coronary artery disease.mp. | 224835 |
| 17 | exp Stroke/ | 193163 |
| 18 | stroke.mp. | 416383 |
| 19 | heart failure.mp. | 371094 |
| 20 | or/12-19 | 3996701 |
| 21 | 7 and 20 | 3816 |
| 22 | 21 not 11 | 3460 |
| 23 | 22 not (congenital heart defects/ or congenital heart disease.mp.) | 3402 |
| 24 | Diabetes Mellitus, Type 2/ | 47097 |
| 25 | type 2 diabetes.tw. | 175976 |
| 26 | insulin/ | 316799 |
| 27 | insulin.mp. | 745385 |
| 28 | Blood Glucose/ | 174292 |
| 29 | blood glucose.tw. | 100318 |
| 30 | homeostatic model assessment.mp. | 3712 |
| 31 | homa.mp. | 27256 |
| 32 | Glycated Hemoglobin A/ | 22129 |
| 33 | hba1c.tw. | 67098 |
| 34 | hemoglobin a1c.tw. | 13801 |
| 35 | or/24-34 | 871698 |
| 36 | 7 and 35 | 1656 |
| 37 | 36 not metabolic syndrome.mp. | 1495 |
| 38 | 37 not (11 or 23) | 1077 |
| 39 | cohort studies/ | 358969 |
| 40 | longitudinal studies/ | 108540 |
| 41 | follow-up studies/ | 977820 |
| 42 | prospective studies/ | 374316 |
| 43 | retrospective studies/ | 436571 |
| 44 | (cohort or longitudinal or prospective or retrospective).tw. | 2256629 |
| 45 | (follow* adj2 up).tw. | 1506109 |
| 46 | 39 or 40 or 41 or 42 or 43 or 44 or 45 | 3752230 |
| 47 | 11 and 46 | 348 |
| 48 | 23 and 46 | 937 |
| 49 | 38 and 46 | 296 |
| 50 | (Light physical activity determined by a motion sensor decreases insulin resistance, improves lipid homeostasis and reduces visceral fat in high-risk subjects: PreDiabEx study RCT).m_titl. | 1 |
| 51 | (Relationship between baseline physical activity assessed by pedometer count and new-onset diabetes in the NAVIGATOR trial).m_titl. | 1 |
| 52 | 7 and metabolic syndrome.mp. | 368 |
| 53 | 46 and 52 | 85 |
| 54 | 53 not (47 or 48 or 49) | 7 |
| 55 | limit 54 to english language | 6 |
| 56 | remove duplicates from 55 | 6 |
| 57 | (20181* or 2019*).ez. | 0 |
| 58 | 47 and 57 | 0 |
| 59 | 48 and 57 | 0 |
| 60 | 49 and 57 | 0 |
| 61 | 54 and 57 | 0 |
| 62 | 58 or 59 or 60 or 61 | 0 |
| 63 | (20181* or 2019*).dc. | 1687587 |
| 64 | 47 and 63 | 54 |
| 65 | 48 and 63 | 154 |
| 66 | 49 and 63 | 32 |
| 67 | 54 and 63 | 1 |

**Database(s):** CINAHL (Ebscohost)

**Dates: I**nception to August 1, 2019

**Outcome: All-cause Mortality**

( (step w3 count*) or pedometer* or acceleromet* or actigraph* ) AND mortality AND ( (cohort or longitudinal or prospective or retrospective or (follow* w2 up) )

Limiters: Exclude MEDLINE records

**Outcome: CVD**

( (step w3 count*) or pedometer* or acceleromet* or actigraph* ) AND ( cardiovascular disease* or coronary heart disease* or ischemic heart disease or coronary artery disease or stroke or heart failure or metabolic sybdrome ) AND ( (cohort or longitudinal or prospective or retrospective or (follow* w2 up) )

Limiters: Exclude MEDLINE records

**Outcome: Dysglycemia**

( (step w3 count*) or pedometer* or acceleromet* or actigraph* ) AND ( Diabetes or insulin or blood glucose or homeostatic model assessment or homa or “Glycated Hemoglobin A” or hba1c or “hemoglobin a1c” ) AND ( (cohort or longitudinal or prospective or retrospective or (follow* w2 up) )

Limiters: Exclude MEDLINE records

**Database(s):** Cochrane Database of Systematic Reviews and Cochrane Central Register of Controlled Trials

**Dates:** Inception to August 2019

**Outcome: All-cause Mortality**

*Cochrane Database of Systematic Reviews*

Search terms: '(step near/3 count*) or pedometer* or acceleromet* or actigraph* in Title Abstract Keyword AND mortality in Title Abstract Keyword AND cohort or longitudinal or prospective or retrospective or (follow* near/2 up) in Title Abstract Keyword - in Cochrane Reviews, Cochrane Protocols, Trials (Word variations have been searched)'

*Cochrane Central Register of Controlled Trials*

Search terms: '(step near/3 count*) or pedometer* or acceleromet* or actigraph* in Title Abstract Keyword AND mortality in Title Abstract Keyword AND cohort or longitudinal or prospective or retrospective or (follow* near/2 up) in Title Abstract Keyword - in Cochrane Reviews, Cochrane Protocols, Trials (Word variations have been searched)'

**Outcome: CVD**

*Cochrane Database of Systematic Reviews*

Search terms: '(step near/3 count*) or pedometer* or acceleromet* or actigraph* in Title Abstract Keyword AND cardiovascular disease* or coronary heart disease* or ischemic heart disease or coronary artery disease or stroke or heart failure in Title Abstract Keyword AND cohort or longitudinal or prospective or retrospective or (follow* near/2 up) in Title Abstract Keyword - in Cochrane Reviews, Cochrane Protocols, Trials (Word variations have been searched)'

*Cochrane Central Register of Controlled Trials*

Search terms: '(step near/3 count*) or pedometer* or acceleromet* or actigraph* in Title Abstract Keyword AND cardiovascular disease* or coronary heart disease* or ischemic heart disease or coronary artery disease or stroke or heart failure or metabolic syndrome in Title Abstract Keyword AND cohort or longitudinal or prospective or retrospective or (follow* near/2 up) in Title Abstract Keyword - in Cochrane Reviews, Cochrane Protocols, Trials (Word variations have been searched)'

**Outcome: Dysglycemia**

*Cochrane Central Register of Controlled Trials*

Search terms: '(step near/3 count*) or pedometer* or acceleromet* or actigraph* in Title Abstract Keyword AND Diabetes or insulin or blood glucose or homeostatic model assessment or homa or “Glycated Hemoglobin A” or hba1c or “hemoglobin a1c” in Title Abstract Keyword AND cohort or longitudinal or prospective or retrospective or (follow* near/2 up) in Title Abstract Keyword - in Cochrane Reviews, Cochrane Protocols, Trials (Word variations have been searched)'

**Supplementary Table 1. Study Quality Assessment Tool**

| Scored: 1=yes/present; 0=no/unclear/not reported |
| --- |
| 1. Was the study purpose clearly stated? |
| 2. Were eligibility criteria, and the sources and methods of selection of participants clearly deﬁned? |
| 3. Were all outcomes, exposures, predictors, potential confounders, and effect modiﬁers clearly deﬁned using standardized methods of acceptable quality? |
| 4. Was exposure (daily step counts) measurement carried out using standardized methods and with acceptable quality (e.g., number of valid days)? |
| 5. Results: Report the # of individuals at each stage of the study. |
| 6. Descriptive results: give characteristics of study participants (demographic, clinical, social) and information on exposures and potential confounders. |
| 7. Were all statistical methods, including those used to control for confounding and to examine subgroups and interactions, clearly described? |
| 8. Were methods for dealing with missing data presented? |
| 9. Were results from other analyses (sensitivity analyses, interactions, subgroups) reported? |
| 10. Were unadjusted estimates and, if applicable, confounder-adjusted estimates and their precision (e.g. 95% conﬁdence interval) reported? |
| 11. Were study limitations clearly stated? |

**Supplementary Table 2. Description of analysis in included studies, by outcome category.**

| **Outcome category**  **(number of studies)** | **Reference** | **Analysis method** | **Variables adjusted for in the analysis** | | | **Daily step count value(s) in analysis** |
| --- | --- | --- | --- | --- | --- | --- |
|  |  |  | **Sociodemographic** | **Medical history or biomarkers** | **Behavior** |  |
| **All-cause mortality**  **(N=5)** | Dwyer, 2015 | Cox proportional hazard regression | Age, sex, BMI, education, study cohort |  | Diet, alcohol consumption, smoking status, total energy intake | Continuous (per 1000 steps/day) |
|  |  |  |  |  |  | Quintiles (≤5500, 5551-8000, 8001-10,000, 10,001-13,500, and 13,501-39,164 steps/day at baseline) |
|  |  |  |  |  |  | Change in steps/day analysis: any increase in steps/day vs no increase |
|  | Fox, 2015 | Cox proportional hazard regression | Age, sex, education, Index of Multiple Deprivation, weight status, lower limb function | Self-reported number of chronic diseases |  | Continuous (per 1000 steps/day) |
|  |  |  |  |  |  | Tertiles (<3196, 3196-5170, and >5170 steps/day) |
|  | Jefferis, 2018 | Cox proportional hazard regression | Age, region of residence, social class, living alone, BMI | Mobility disability | Wear time, season of wear, sleep duration, smoking status, alcohol consumption | Continuous (per 1000 steps/day) |
|  |  |  |  |  |  | Quartiles (121-2927, 2928-4532, 4533-6412, 6413-17781 steps/day) |
|  | Lee, 2019 | Cox proportional hazard regression | Age, BMI | Hormone therapy, parental history of MI, family history of cancer, general health, history of CVD, cancer, hypertension, high cholesterol, diabetes; cancer screening | Wear time, smoking status, alcohol use, intakes of saturated fat, fiber, fruits, and vegetables | Continuous (per 1,000 steps/day) |
|  |  |  |  |  |  | Quartiles (2718, 4363, 5905, 8442 median steps/day, respectively) |
|  | Yamamoto, 2018 | Cox proportional hazard regression | Sex, BMI | Number of medications | Smoking status, alcohol intake | Continuous (per 1,000 steps/day) |
|  |  |  |  |  |  | Quartiles (<4503, 4503-6110, 6111-7971, >7972) |
| **CVD events**  **(N=4)** | Cochrane, 2017 | Cox proportional hazard regression | Randomization, site, sex, race, age, education, marital status, living alone | Cardiovascular disease history, diabetes mellitus, antihypertensive use, PSQI, blood pressure | Wear time | Continuous (per 500 steps/day) |
|  | Huffman, 2014 | Linear regression | Age, sex, race, geographic region, | Family history of diabetes, aspirin use, antihypertensive use, lipid-lowering agent | Smoking status | Continuous (per 2000 steps/day) |
|  | Jefferis, 2019 | Cox proportional hazard regression | Age, region of residence, social class, living alone, BMI | Mobility disability | Wear time, season of wear, sleep duration, smoking status, alcohol consumption | Continuous (per 1000 steps/day) |
|  |  |  |  |  |  | Quartiles (121-2943, 2944-4540, 4541-6406, 6407-17781 steps/day) |
|  | Yates, 2014 | Cox proportional hazard regression | Randomization, BMI, age, region, sex | Coronary heart disease composite, cerebrovascular composite, significant abnormal electrocardiogram, insignificant abnormal ECG, albumin/creatinine ratio, pulmonary composite, peripheral artery disease composite congestive heart failure, chronic obstructive pulmonary disease, pulse pressure, temporary atrial fibrillation or flutter, estimated glomerular filtration rate, hemoglobin, LDL-cholesterol, and antihypertensive medication use | Smoking status, sodium intake | Continuous (per 2,000 steps/day)  *Baseline steps/day truncated at 20,000 |
|  |  |  |  |  |  | Change in steps/day analysis: Change from baseline to 12 months (per 2000 steps/day difference in change). Change truncated at 10,000 steps/day. |
| **Dysglycemia**  **(N=8)** | Kraus, 2018 | Cox proportional hazard regression | Age, sex, region, BMI, | Blood pressure, coronary heart disease composite, fasting glucose, 2-hour glucose on oral glucose tolerance test, hemoglobin A1C, LDL, HDL, platelet count, hemoglobin concentration |  | Continuous (per 2000 steps/day). Steps/day truncated at 10,000. |
|  | Ponsonby, 2011 | Logistic regression | Age, sex |  | Baseline steps/day | Continuous (per 1,000 steps/day) |
|  | Dwyer, 2011 | Linear regression | Age, sex, BMI at follow-up, waist-to-hip ratio, SES, education | HOMA insulin sensitivity at baseline | Baseline steps/day, total energy intake, alcohol consumption, smoking status, length of follow-up | Continuous (per 1000 steps/day) |
|  | Herzig, 2014 | Compared least and most active quartiles using Dunnett’s test |  | Body weight change |  | Quartiles (1780-2810, 2940-4010, 4010-6020 and 6520-21 000) |
|  | Siddiqui, 2018 | Pearson’s correlation coefficients |  |  |  | N/A |
|  | Tudor-Locke, 2004 | Spearman correlation coefficients |  |  |  | N/A |
|  | Van Dyck, 2013 | ANCOVAs | Age | Years since diagnosis, insulin use, change scores between pre- and post-measurements in total cholesterol and triglycerides | Baseline steps/day | Change in steps/day: increase ≥4,000 steps/day vs no increase |
|  | Yates, 2015 | Generalized estimating equations | Age, randomization, sex, race, region, waist circumference | Glucose history in preceding 3 years, systolic blood pressure, pulse pressure, family history of diabetes, chronic obstructive pulmonary disease, atrial fibrillation/flutter, LDL and HDL cholesterol, platelets, hemoglobin, log of albumin/creatinine ratio, sodium, estimated GFR, electrocardiogram abnormalities, congestive heart failure, cerebrovascular disease, coronary heart disease, pulmonary disease, peripheral artery disease | Smoking status | Change in log steps/day t_-2_ (2 years prior to glucose test) to t_-1_ (1 year prior to glucose test) and log steps/day at t_-2_ |

**Supplementary Table 3. Quality Criteria Index Scores of Included Studies.**

|  | Dwyer, 2015 | Fox, 2015 | Jefferis, 2018 | Lee, 2019 | Yamamoto, 2018 | Cochrane, 2017 | Huffman, 2014 | Jefferis, 2019 | Yates, 2014 | Kraus, 2018 | Ponsonby, 2011 | Dwyer, 2011 | Herzig, 2014 | Siddiqui, 2018 | Tudor-Locke, 2004 | Van Dyck, 2013 | Yates, 2015 |
| --- | --- | --- | --- | --- | --- | --- | --- | --- | --- | --- | --- | --- | --- | --- | --- | --- | --- |
| **Quality Score** | **6** | **9** | **7** | **9** | **9** | **6** | **8** | **8** | **10** | **10** | **7** | **9** | **7** | **6** | **6** | **7** | **9** |
| 1. Study purpose | 1 | 1 | 0 | 1 | 1 | 0 | 1 | 1 | 1 | 1 | 1 | 1 | 1 | 1 | 1 | 1 | 1 |
| 2. Eligibility criteria, sources and methods of participant selection | 0 | 1 | 0 | 1 | 1 | 1 | 1 | 1 | 1 | 1 | 1 | 1 | 1 | 1 | 1 | 1 | 1 |
| 3. Outcomes, exposures, predictors, potential confounders, and effect modiﬁers clearly deﬁned using standardized methods | 0 | 1 | 1 | 1 | 1 | 1 | 1 | 1 | 1 | 1 | 1 | 1 | 1 | 1 | 1 | 1 | 1 |
| 4. Exposure measurement carried out using standardized methods | 0 | 1 | 0 | 1 | 0 | 0 | 0 | 0 | 0 | 0 | 0 | 0 | 1 | 1 | 1 | 1 | 1 |
| 5. All statistical methods clearly described | 1 | 1 | 1 | 1 | 1 | 1 | 1 | 1 | 1 | 1 | 1 | 1 | 0 | 1 | 0 | 1 | 1 |
| 6. Methods dealing with missing data appropriate | 0 | 0 | 0 | 1 | 0 | 0 | 0 | 0 | 1 | 1 | 0 | 0 | 0 | 0 | 0 | 0 | 0 |
| 7. Report the number of individuals at each stage of the study | 1 | 1 | 1 | 1 | 1 | 0 | 1 | 1 | 1 | 1 | 1 | 1 | 1 | 0 | 1 | 1 | 1 |
| 8. Give characteristics of study participants and information on exposures and potential confounders | 0 | 1 | 1 | 0 | 1 | 1 | 1 | 1 | 1 | 1 | 1 | 1 | 1 | 0 | 1 | 1 | 1 |
| 9. Results from other analyses reported | 1 | 0 | 1 | 1 | 1 | 0 | 0 | 1 | 1 | 1 | 0 | 1 | 0 | 1 | 0 | 0 | 0 |
| 10. Unadjusted and adjusted estimates and their precision reported | 1 | 1 | 1 | 1 | 1 | 1 | 1 | 1 | 1 | 1 | 1 | 1 | 0 | 0 | 0 | 0 | 1 |
| 11. Study limitations clearly stated | 1 | 1 | 1 | 1 | 1 | 1 | 1 | 1 | 1 | 1 | 0 | 1 | 1 | 0 | 0 | 0 | 1 |
